# The Impact of Recombination on Nucleotide Substitutions in the Human Genome

**DOI:** 10.1371/journal.pgen.1000071

**Published:** 2008-05-09

**Authors:** Laurent Duret, Peter F. Arndt

**Affiliations:** 1 Laboratoire de Biométrie et Biologie Evolutive, Université de Lyon, Université Lyon 1, CNRS, UMR 5558, Villeurbanne, France; 2 Department for Computational Molecular Biology, Max Planck Institute for Molecular Genetics, Berlin, Germany; University of Chicago, United States of America

## Abstract

Unraveling the evolutionary forces responsible for variations of neutral substitution patterns among taxa or along genomes is a major issue for detecting selection within sequences. Mammalian genomes show large-scale regional variations of GC-content (the isochores), but the substitution processes at the origin of this structure are poorly understood. We analyzed the pattern of neutral substitutions in 1 Gb of primate non-coding regions. We show that the GC-content toward which sequences are evolving is strongly negatively correlated to the distance to telomeres and positively correlated to the rate of crossovers (R^2^ = 47%). This demonstrates that recombination has a major impact on substitution patterns in human, driving the evolution of GC-content. The evolution of GC-content correlates much more strongly with male than with female crossover rate, which rules out selectionist models for the evolution of isochores. This effect of recombination is most probably a consequence of the neutral process of biased gene conversion (BGC) occurring within recombination hotspots. We show that the predictions of this model fit very well with the observed substitution patterns in the human genome. This model notably explains the positive correlation between substitution rate and recombination rate. Theoretical calculations indicate that variations in population size or density in recombination hotspots can have a very strong impact on the evolution of base composition. Furthermore, recombination hotspots can create strong substitution hotspots. This molecular drive affects both coding and non-coding regions. We therefore conclude that along with mutation, selection and drift, BGC is one of the major factors driving genome evolution. Our results also shed light on variations in the rate of crossover relative to non-crossover events, along chromosomes and according to sex, and also on the conservation of hotspot density between human and chimp.

## Author Summary

Mammalian genomes show a very strong heterogeneity of base composition along chromosomes (the so-called isochores). The functional significance of these peculiar genomic landscapes is highly debated: do isochores confer some selective advantage, or are they simply the by-product of neutral evolutionary processes? To resolve this issue, we analyzed the pattern of substitution in the human genome by comparison with chimpanzee and macaque. We show that the evolution of base composition (GC-content) is essentially determined by the rate of recombination. This effect appears to be much stronger in male than in female germline, which rules out selective explanations for the evolution of isochores. We show that this impact of recombination is most probably a consequence of the process of biased gene conversion (BGC). This neutral process mimics the action of selection and can induce strong substitution hotspots within recombination hotspots, sometimes leading to the fixation of deleterious mutations. BGC appears to be one of the major factors driving genome evolution. It is therefore essential to take this process into account if we want to be able to interpret genome sequences.

## Introduction

Genomic landscapes are not uniform across vertebrate chromosomes. Notably, the genomes of amniotes (mammals, birds and reptiles) show a very strong heterogeneity of base composition along chromosomes (the so-called isochores) (for review, [1]). These Mb-scale variations in GC-content result from variations of substitution patterns that have affected both coding and non-coding regions. These genomic landscapes are correlated with many other important features (gene density, intron size, distribution of transposable elements, replication timing). Thus, isochores clearly reflect some fundamental aspects of genome organization. Although isochores have been discovered more than 30 years ago [2], the reason for their origin is still highly debated: are they the result of selection [3]–[8], or do they simply reflect variations in neutral substitution patterns [9]–[15]?

Unraveling the origin of isochores (neutral evolution or selection) is essential to understand the functional significance (if any) of this peculiar genomic organization. Moreover, a better knowledge of genome-wide variations in neutral evolutionary processes is also important for practical reasons. Indeed, comparative sequence analysis is commonly used to identify genes or regulatory elements within genomes. The basic principle of this approach is that functional elements are subject to the action of natural selection and therefore, their pattern of sequence variation (within populations or between different species) differs from what would be expected under the null hypothesis of neutral evolution. Hence, to be able to detect functional elements within genomes it is crucial to understand the parameters that affect the neutral processes of sequence evolution.

Recently, different lines of evidence have suggested that isochores might be a consequence of the process of recombination (for review, [16]). Notably, analyses of the pattern of substitution in primate non-coding sequences have shown that recombination affects the relative rate of AT→GC and GC→AT substitutions [15],[17],[18]. We and others have proposed that this effect might result from the neutral process of biased gene conversion (BGC) [11],[14],[19],[20]. According to this model, gene conversion (i.e. the copy/paste during meiotic recombination of one allele onto the other one at heterozygous loci) is biased in favor of GC-alleles, which leads to an increase of probability of fixation of GC-alleles compared to AT-alleles. Thus, BGC should lead to an enrichment in GC-content in genomic regions of high recombination compared to regions of low recombination. Understanding the impact of BGC on genome evolution is of fundamental importance. Indeed, the effect of BGC is very similar to that of directional selection [21], and hence BGC can confound the tests that have been developed to detect selection in genomic sequences [22].

Although many lines of evidence support the BGC hypothesis [16], there remain several important theoretical problems with this model, pointed out by Spencer and colleagues [23]. First, it is now clearly established that in humans, recombination occurs predominantly in hotspots (typically 2 kb long) that cover about 3% of the genome [24]. If recombination affects only very short regions, how can it drive the evolution of GC-content in Mb-long genomic fragments? Second, the analysis of human SNPs has shown that there is a fixation bias in favor of GC-alleles (in agreement with the BGC model), but that this bias is relatively weak [23]. Furthermore, the location of recombination hotspots is not conserved between human and chimpanzee, which indicates that hotspots have a short lifespan [25],[26]. Given these spatial and temporal fluctuations in recombination rate, is it possible that the BGC process affects the evolution of base composition?

Some other authors have proposed that it is the base composition of sequences (and not recombination) that is the major determinant of substitution patterns [13]. Indeed, the rate of cytosine mutation depends directly on the DNA melting (and hence on the GC-content of sequences). Therefore, the GC-content is expected to affect the relative rate of AT→GC and GC→AT substitutions. Given that GC-content and recombination rate are positively correlated, this effect could contribute to the correlations between recombination rate and substitution patterns that were previously reported [15],[17],[18].

To address these issues we performed two complementary analyses. First, we took advantage of newly available data (fine scale crossover map in humans and complete genome sequences of human, chimpanzee and macaque) to re-assess the genome-wide relationship between patterns of substitution and recombination, controlling for the impact of GC-content. For this purpose, we developed a new method to compute substitution rates for individual nucleotides, taking into account the hypermutability of CpG dinucleotides and the non-stationarity of base composition. This method is based on a maximum-likelihood (ML) approach, and hence is more reliable than the parsimony approach used previously. Second, we modeled the process of BGC, taking into account recombination hotspots, to theoretically assess the potential impact of this molecular drive on the evolution of genome landscapes.

Our analyses confirm that recombination is the major determinant of the evolution of GC-content and allows us to definitively reject selectionist models of isochore evolution. Moreover, these analyses shed light on the evolution of recombination rate since the divergence between human and chimpanzee, on the distribution of non-crossover recombination events and on the differences in patterns of recombination between males and females. Finally, theoretical calculations demonstrate that despite the short lifespan of recombination hotspots, BGC can have a strong impact on genome evolution.

## Results

The present base composition of a genomic fragment reflects the average pattern of substitutions to which it has been exposed during evolutionary times. Thus, to better understand the evolutionary forces that have been responsible for the strong regional variations in base composition along mammalian genomes (the isochores), we studied the pattern of substitution in the human lineage, by comparison with chimpanzee and using macaque as an outgroup to orientate changes. Patterns of substitutions were computed in non-overlapping windows of 1 Mb, sliding along human chromosomes.

We analyzed 1 Gb of non-coding sequences (introns or intergenic regions). Functional non-coding sequences constitute only a very small fraction of mammalian genomes [27],[28]. Hence, non-coding sequences can be assumed to evolve essentially neutral, not constrained by natural selection. The evolution of sexual chromosomes differs from that of autosomes, because of differences in recombination rate, effective population sizes and mutation rates [29]. We therefore analyzed the X chromosome separately from the rest of the genome (we could not analyze the Y chromosome because it has not been sequenced in macaque).

### A New Method To Infer Substitution Rates Accounting for CpG Hypermutability and Non-Stationarity

In previous works, we had used parsimony to infer substitutions [15],[18]. While this concept is very simple and powerful for closely related sequences, it fails as divergence among sequences increases [30],[31]. Notably, because of CpG mutation hotspots, parsimony may fail at reconstructing sequences of the human/chimp last common ancestor [16]. Hence, we had to exclude from our analyses many sites for which the ancestral state was ambiguous [15],[18]. One can avoid such problems using the maximum likelihood approach, which was pioneered by Felsenstein [32]. In this framework one searches the parameters of the substitution rate matrix that maximizes the likelihood of sequence data given a stochastic model of nucleotide substitutions. However the various ML methods to phylogeny reconstruction that have been proposed previously, make at least one of the following assumptions: (i) the substitution model is time-reversible and the same in all branches of a given tree (only the branch length might vary from one branch to another, not all substitution processes are considered independently), (ii) the genomes under considerations are in the stationary state with respect to this model, and (iii) neighbor dependent nucleotide substitutions can be neglected. These assumption are thought to be necessary to efficiently compute the likelihood for a given substitution model and tree topology [32]. However all these simplifying assumptions are not necessarily granted: notably, we know that the base composition is by far not constant and stationary for mammalian species [15], [16], [33]–[38]. Moreover, the neighbor dependent and irreversible CpG methylation deamination process (CpG→CpA/TpG) is the predominant nucleotide substitution process in vertebrates [35],[39],[40]. We introduce here a new ML method, that takes into account non-stationary and non-reversible processes (as already proposed [41],[42]) and furthermore includes neighbor dependent substitutions processes, like the CpG methylation deamination process. This approach is described in detail in the methods section.

We measured 7 substitution rates (pooling together complementary rates): the 4 transversion rates (A→C+T→G; A→T+T→A; C→A+G→T; C→G+G→C), the 2 transition rates at non-CpG sites (A→G+T→C; G→A+C→T), and the transition rate at CpG sites (G→A+C→T). We will hereafter use the notation 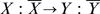 to indicate complementary substitutions (e.g. A:T→G:C = A→G+T→C). When convenient, we will use the notation W (weak) for A or T and S (strong) for C or G. Thus, the notation W→S indicates all substitutions (transitions or transversions) from A or T to G or C.

Note that the total substitution rate (*K*) in a given genomic regions depends on its base composition and on the base-specific substitution rates. In the model considered here (with 7 base-specific substitution rates) *K* is given by the following equation:
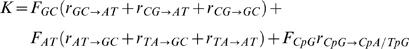
(1)where *F_GC_, F_AT_* and *F_CpG_* denote the frequencies of the different categories of sites and the parameters 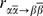 denote the base-specific substitution rates.

We measured base-specific substitution rates independently in the human and chimpanzee lineages. From these substitution rates, we inferred for each lineage the stationary GC-content of sequences (hereafter noted GC*), using a method that accounts for CpG hypermutability [43]. GC* corresponds to the GC-content that sequences would reach at equilibrium if patterns of substitution remained constant over time. GC* therefore provides information about the recent trend of evolution of GC-content. In fact, GC* can be considered as a summary statistics of the average substitution matrix during the last 6 Myrs. It should be noticed that GC* is a measure of substitution patterns that is independent of the total substitution rate; it simply reflects the relative contribution of W→S and S→W substitutions to the total number of substitutions.

### Impact of GC-Content and Crossover Rate on Substitution Patterns

We first investigated the relationship between GC*, recombination rate and the regional base composition (GC-content). As an estimator of recombination rate, we took the rate of crossover from the HAPMAP genetic map [44] and from the deCODE genetic map [45]. The HAPMAP genetic map is based on patterns of allelic associations, and hence reflects the sex-averaged crossover rate that occurred in human populations (i.e. the historical crossover rate). The deCODE genetic map is based on pedigree studies and provides both sex-averaged and sex-specific crossover rates.

**Figure 1:**
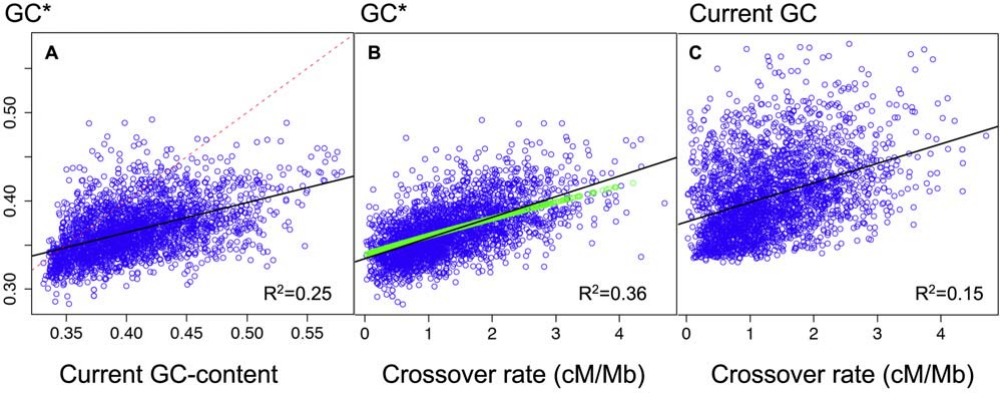
Correlations between the stationary GC-content (GC*), the current GC content and the crossover rate in human autosomes. Each dot corresponds to a 1 Mb-long genomic region. (A) GC* *vs.* current GC-content. The dashed line indicates the slope 1. (B) GC* *vs.* crossover rate (HAPMAP). Green dots correspond to the predictions of the BGC model (model M1, *N* = 10,000) (C) Current GC-content *vs.* crossover rate. Regression lines and Pearson's correlation R^2^ are indicated.

In agreement with our previous results [15], we found at the 1 Mb scale a strong correlation between GC* and the sex-averaged rate of crossover on autosomes, both with the HAPMAP data (Pearson correlation R^2^ = 0.36, Figure 1b) and with the deCODE data (R^2^ = 0.31). GC* is also strongly correlated with the local GC-content (R^2^ = 0.25, Figure 1a), but this correlation is weaker than with the crossover rate. We observed that the pattern of substitution tends to decrease the GC-content of our genome: GC* is lower than the present GC, particularly in GC-rich regions (Figure 1a). However note that this process is extremely slow: since the divergence between human and chimpanzee (about 6 Myrs ago), regions with more the 50% GC lost about 0.2% GC. If these substitution patterns would not change in time, we can extrapolate that it would take at least 500 Myrs for such a region to reach a GC-content of 40%. Thus, the human genome appears to be evolving toward a more homogenous and less GC-rich base composition, in agreement with previous findings [15], [16], [33]–[38].

It should be noted that the correlation between GC* and the current GC is far from perfect (75% of the variance in GC* is not predicted by the current GC-content). In other words, the GC-content toward which sequences are evolving is largely independent from the current GC-content. Thus, the forces that have driven the evolution of isochores in mammalian genomes have changed both in intensity (these forces are not strong enough to maintain GC-rich isochores) and in localization along chromosomes.

GC* correlates strongly both with crossover rate and GC-content. We have previously proposed that recombination was the major determinant of GC* [15]. However, other authors also suggested that the GC-content was a strong direct determinant of GC*, because the rate of cytosine mutation depends directly on the DNA melting (and hence on the GC-content of sequences) [13]. Given that GC-content and crossover rate are also positively correlated (R^2^ = 0.15, Figure 1c), this raises the question of which variables (GC, recombination or both) are truly involved in determining GC*, and which happen to covary simply because they are influenced by another, causal variable. It has been proposed that a higher GC-content might promote recombination [46]–[48]. Indeed, in human, recombination hotspots occur preferentially in locally GC-rich regions [23]. Thus, if GC-content determines both the recombination rate and GC*, this could explain the correlation between the rate of crossover and GC*. However, in agreement with our previous analyses [15], we found that the rate of crossover correlates much more strongly with the stationary GC-content (GC*) than with the present GC-content (GC) (compare Figure 1b and 1c): the crossover rate explains 36% of the variance in GC*, compared to only 15% of the variance in GC. The same pattern is observed on the X chromosome (Table 1). If the correlation between GC* and crossover rate was due to the impact of base composition on recombination, then we would have expected a much stronger correlation of the rate of crossover with the present GC-content than with the stationary GC-content (i.e. the future GC-content of sequences). Our observations therefore definitively demonstrate that at the genomic scale considered here (1 Mb), recombination drives the evolution of GC-content.

**Table 1:** Correlation between the crossover rate and the current GC-content or the stationary GC-content (GC*), and correlations between human and chimp GC*.

Sequence type	Tiling	Human crossover rate *vs.*			GC* human *vs.*
	(Mb)	Current GC	GC* human	GC* chimp	GC* chimp
		R^2^	R^2^	R^2^	R^2^
Non-coding	10	0.30	0.61	0.56	0.81
(autosomes)	5	0.21	0.55	0.50	0.78
	2	0.18	0.47	0.47	0.76
	1	0.15	0.36	0.36	0.70
	0.5	0.12	0.27	0.26	0.60
	0.2	0.09	0.15	0.15	0.43
	0.1	0.04	0.06	0.06	0.27
Intergenic	1	0.13	0.30	0.29	0.59
Introns	1	0.16	0.28	0.29	0.53
Non-coding (X)	1	0.01 (a)	0.17 (b)	0.07 (c)	0.66

Crossover rate: HAPMAP. Pearson's correlations (R^2^) are given for different window sizes, and different genomic regions (all non-coding sequences or introns and intergenic regions), for autosomes (A) and for the X chromosome. All correlations have a p-value <10^−10^, except (a) non-significant, (b) p-value = 3 10^−5^ and (c) p-value = 7 10^−3^.

This does not exclude however that the GC-content might also affect GC*. Indeed, multivariate regression indicate that both GC-content and crossover rate are significant predictors of GC* (p<10^−10^). Thus, the correlation between GC* and GC is not simply an indirect consequence of the correlation between GC and crossover rate. Taken together, GC and crossover rate explain 44% of the variance of GC*.

We investigated the correlation between crossover rate and GC* separately in introns and intergenic regions. We found similar correlations for all kinds of non-coding sequences (Table 1), which indicates that recombination affects the evolution of base composition in all genomic compartments, transcribed or not.

### The Impact of Recombination on Substitution Patterns Is Underestimated

HAPMAP and deCODE sex-averaged crossover rates are not perfectly correlated (R^2^ = 0.53 at the 1 Mb scale), which indicates that these data are noisy. It is presently not known to which extent this noise is due to the imprecision of the methods used to estimate crossover rates or to real variations in crossover rates during the evolution of human populations (given that recombination rates evolve rapidly, crossover rates estimated from pedigree-based genetic maps may differ from historical crossover rates). But in any case, this indicates that HAPMAP and deCODE crossover rates are not perfect predictors of the average recombination rate in the human lineage during the last 6 Myrs. Thus, even if recombination was the unique determinant of GC*, we would not expect a perfect correlation between GC* (which is inferred from the pattern of substitutions in the human lineage during the last 6 Myrs) and the HAPMAP or deCODE crossover rates. Taken together, HAPMAP and deCODE sex-averaged crossover rates explain 39% of the variance in GC* (i.e. significantly more than each variable taken separately, p<10^−10^). However, this is certainly still an underestimate of the true correlation between GC* and recombination rate.

### Patterns of Substitution Vary with the Distance to Telomeres

**Figure 2:**
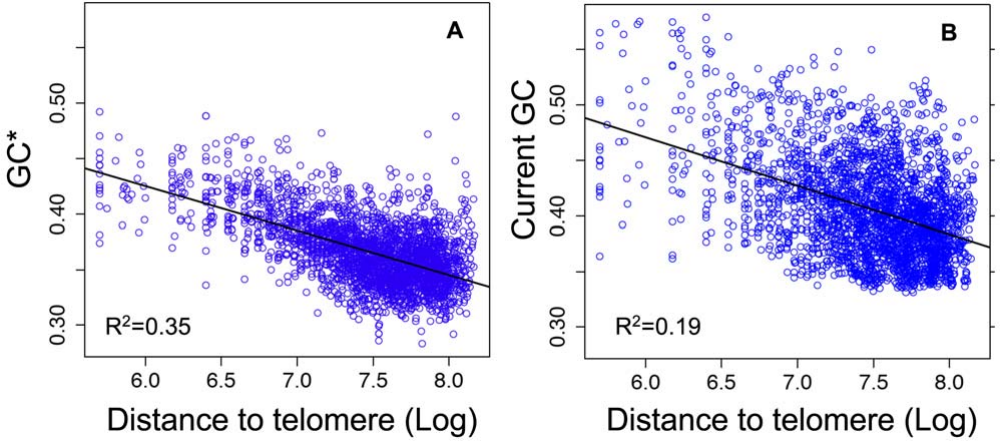
Correlations between the stationary GC-content (GC*), the current GC content and the distance to telomeres in human autosomes. Each dot corresponds to a 1 Mb-long genomic region. (A) GC* *vs.* LDT (Log distance to telomere in bp). (B) Current GC-content *vs.* LDT. Regression lines and Pearson's correlation R^2^ are indicated.

To try to better characterize the impact of recombination on sequence evolution, we searched for additional predictors of recombination rate. It is known that in humans, the rate of recombination increases near telomeres [45],[49]. Indeed, there is a negative correlation between HAPMAP crossover rates and the distance to telomere (in log scale, hereafter noted LDT) (R^2^ = 0.27, p<10^−10^). We observed a strong negative correlation between GC* and LDT (R^2^ = 0.35, p<10^−10^) (Figure 2a). As shown above for crossover rates, LDT correlates much more strongly with GC* than with the current GC-content (R^2^ = 0.19, Figure 2b). Again, this demonstrates that the correlation between LDT and GC* is not an indirect consequence of the correlation between LDT and GC.

To try to disentangle the contribution of the different variables (crossover rate, GC-content and LDT) to the variation of GC*, we performed a multivariate regression analysis. By using a stepwise procedure, we found that the best two predictors of GC* are the HAPMAP crossover rates and LDT (Table 2, Supplementary Text S1). Taken together, HAPMAP crossover rate and LDT explain 47% of the variance in GC* at the 1 Mb scale. The GC-content significantly improves the model, but the gain in accuracy of prediction is relatively modest (R^2^ = 0.51, Table 2). The addition of other variables (deCODE sex-averaged, male or female recombination rates) does not further improve the model.

**Table 2:** Partial correlation analysis of the three predictors of stationary GC-content (GC*) and base-specific substitution rates in the human lineage: current GC-content (GC), crossover rate (CO) and the distance to telomeres (LDT).

Variable *X*		Partial correlation						R^2^	R^2^
		*X*,GC|(CO,LDT)		*X*,CO|(GC,LDT)		*X*,LDT|(GC,CO)		*X,*	Full
		R	p	R	p	R	p	(CO,LDT)	model
Stationary GC-content (GC*)		0.30	<10^−10^	0.43	<10^−10^	−0.30	<10^−10^	0.47	0.51
Substitution rates:									
W→S	A:T→G:C	−0.29	<10^−10^	0.39	<10^−10^	−0.35	<10^−10^	0.28	0.35
	A:T→C:G	−0.20	<10^−10^	0.32	<10^−10^	−0.33	<10^−10^	0.25	0.29
S→W	C:G→T:A (CpG)	−0.64	<10^−10^	0.04	NS	−0.10	5 10^−10^	0.05	0.44
	C:G→T:A (non-CpG)	−0.41	<10^−10^	0.10	3 10^−5^	−0.15	<10^−10^	0.00	0.18
	C:G→A:T	−0.52	<10^−10^	0.04	NS	−0.15	<10^−10^	0.01	0.29
W→W	A:T→T:A	−0.40	<10^−10^	0.13	2 10^−8^	−0.18	<10^−10^	0.01	0.17
S→S	C:G→G:C	−0.24	<10^−10^	0.20	<10^−10^	−0.26	<10^−10^	0.11	0.17

The R^2^ estimates of the multivariate regression analysis are indicated for the model including only the 2 predictors of recombination rates (i.e. CO and LDT) and for the full model (including the 3 predictors). Data: autosomes, 1 Mb windows. Crossover rates from HAPMAP. NS: non-significant.

### Impact of GC-Content and Recombination Rate on Base-Specific Substitution Rates in Autosomes

**Figure 3:**
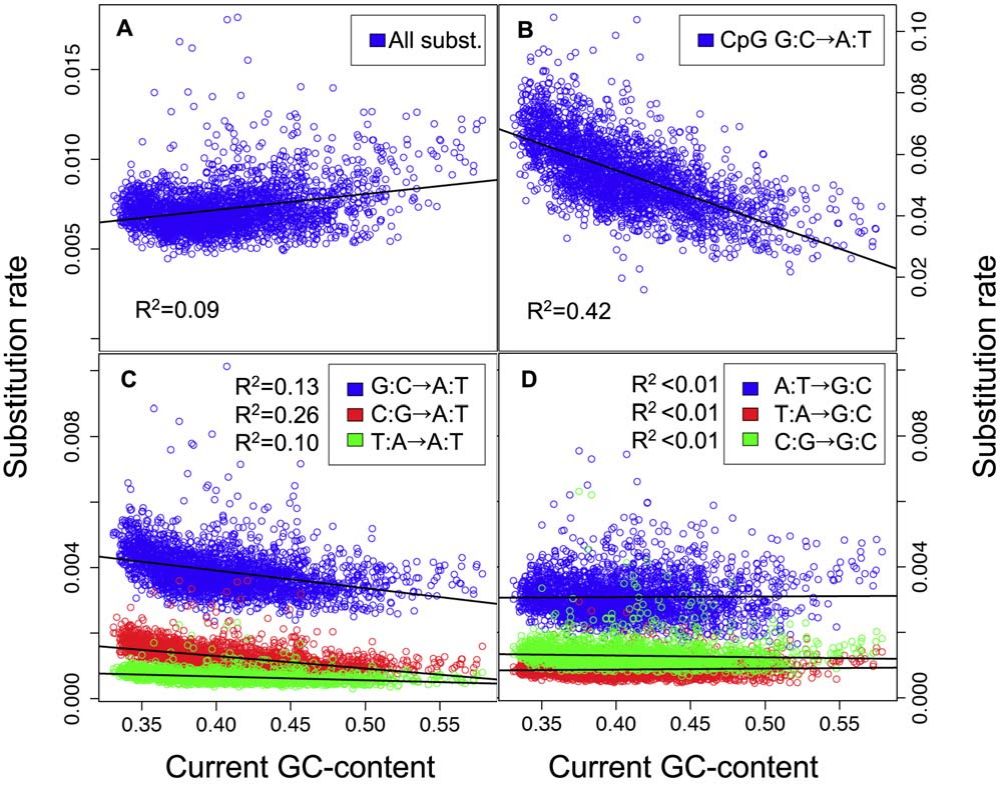
Correlations between substitution rates and the current GC content in human autosomes. Each dot corresponds to a 1 Mb-long genomic region. Substitution rates: number of substitutions per site in the human lineage since the divergence from chimpanzee. (A) Total substitution rate. (B–D) Base-specific substitution rates: (B) CpG G:C→A:T transition rate. (C) non-CpG S→W and W→W substitution rates. (D) W→S and S→S substitution rates. Regression lines and Pearson's correlation R^2^ are indicated.

**Figure 4:**
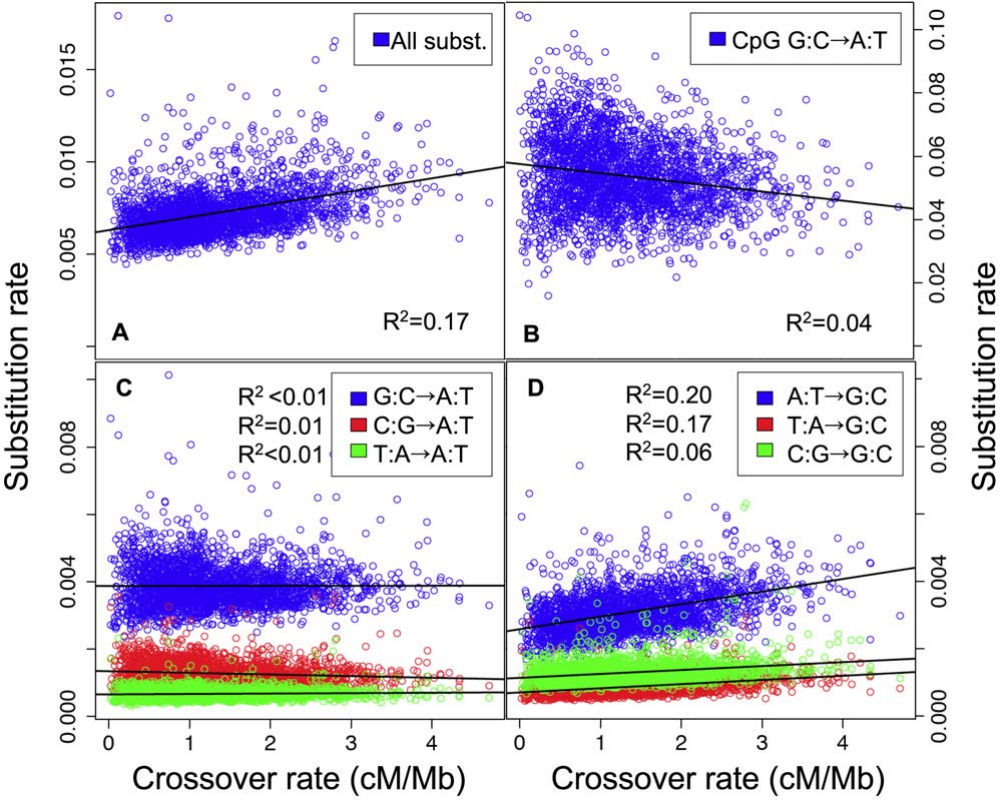
Correlations between substitution rates and crossover rate in human autosomes. Each dot corresponds to a 1 Mb-long genomic region. Substitution rates: number of substitutions per site in the human lineage since the divergence from chimpanzee. (A) Total substitution rate. (B–D) Base-specific substitution rates: (B) CpG G:C→A:T transition rate. (C) non-CpG S→W and W→W substitution rates. (D) W→S and S→S substitution rates. Regression lines and Pearson's correlation R^2^ are indicated.

To get a clearer picture of the dependencies of the stationary GC-content on the recombination rate and GC-content, we analyzed the base-specific substitution rates (which are the underlying determinants of GC*) according to crossover rate, LDT and the current GC-content. Partial correlation analyses indicate that all base-specific substitution rates are affected negatively by the current GC-content and positively by recombination rate (i.e. positively by crossover rate and negatively by LDT), but the strength of correlations with each variable varies greatly among base-specific substitution rates (Table 2). Note that the effect of LDT on base-specific substitution rates is always parallel to that of crossover rate, which supports our assumption that LDT and crossover rate are two complementary predictors of the recombination rate. Interestingly, S→W and W→W substitution rates show a very weak dependency on recombination rate, but a strong dependency on GC-content (compare in Table 2 the R^2^ of the model including only recombination predictors – i.e. LDT and crossover rate - to the R^2^ of the full model). Conversely, W→S substitution rates show a much stronger dependency on recombination rate than on GC-content. This dependency of W→S substitution frequencies on the recombination rates is in the end responsible for the correlation of GC* on the recombination rate. S→S substitution rates appear to be affected by both variables. The fact that base-specific substitution rates are differently affected by GC-content and by recombination rate is clearly seen in pairwise correlation analyses (Table 3; compare Figures 3 and 4).

**Table 3:** Summary of stationary GC-content (GC*) and base-specific substitution rates in the human lineage and their pairwise correlations with the current GC-content (GC), crossover rate (CO) and the distance to telomeres (LDT).

Variables		Average	Pairwise correlations								
			Current GC			CO			LDT		
			Sign	R^2^	p	Sign	R^2^	p	Sign	R^2^	p
Stationary GC-content (GC*)		0.37	+	0.25	<10^−10^	+	0.36	<10^−10^	−	0.35	<10^−10^
Substitution rates:											
W→S	A:T→G:C	0.0031		0.000	NS	+	0.20	<10^−10^	−	0.22	<10^−10^
	A:T→C:G	0.0009	+	0.004	0.002	+	0.17	<10^−10^	−	0.21	<10^−10^
S→W	C:G→T:A (CpG)	0.0540	−	0.42	<10^−10^	−	0.04	<10^−10^	+	0.04	<10^−10^
	C:G→T:A (non-CpG)	0.0039	−	0.13	<10^−10^		0.000	NS		0.000	NS
	C:G→A:T	0.0013	−	0.26	<10^−10^	−	0.01	6 10^−9^	+	0.005	3 10^−4^
W→W	A:T→T:A	0.0007	−	0.10	<10^−10^	+	0.004	0.003	−	0.01	2 10^−5^
S→S	C:G→G:C	0.0013	−	0.005	8 10^−4^	+	0.06	<10^−10^	−	0.10	<10^−10^

Data: autosomes, 1 Mb windows. Crossover rates from HAPMAP. NS: non-significant. R^2^: Pearson's correlation R^2^. The sign of the correlation is indicated (when significantly different from zero).

It should be noticed that the total substitution rate (*K*) is positively correlated to GC-content (Figure 3a). This might seem *a priori* unexpected given that base-specific substitution rates show either a negative correlation (Figure 3b,c) or no correlation with GC-content (Figure 3d). However, *K* depends not only on base-specific substitution rates but also on the base composition (see equation (1)). Thus, given that, S→W substitution rates are on average higher than their respective W→S back substitutions (Table 3), *K* tends to increase with the GC-content (*F_GC_* in equation (1)). In other words, the positive correlation between the total substitution rate and GC-content does not reflect a higher exposure of GC-rich regions to mutagenic factors, but simply a higher proportion of GC bases that are more prone to substitutions than AT bases.

### Conservation of Recombination Rates between Human and Chimpanzee

Given the strong correlation between GC* and recombination rate, GC* can be used as an indicator to investigate the evolution of patterns of recombination. Notably, it is presently not clear what is the time scale and genomic scale of evolution of recombination rate. It has been recently shown that recombination hotspots evolve very rapidly. Indeed, the locations of recombination hotspots in human and chimpanzee are totally uncorrelated, despite considerable sequence identity [25],[26], and it has been demonstrated that hotspot activity may vary strongly among individuals in human populations [50]. Given our previous results, these rapid changes in fine scale recombination maps are expected to lead to variations in substitution patterns during time. In apparent contradiction with that prediction, at the genomic scale considered here (1 Mb), we found a strong conservation of substitution patterns between human and chimpanzee lineages: the correlation between GC* measured in human and chimpanzee orthologous regions is R^2^ = 0.70 (p<10^−10^). Notably, GC* measured in the chimpanzee lineage is more strongly correlated to the rate of crossover measured in human populations (R^2^ = 0.36, i.e. as strong as the correlation observed with human GC*), than to the current GC-content in chimpanzee (R^2^ = 0.24). The only possible interpretation for this correlation is that at the Mb scale, rates of recombination are highly conserved between human and chimpanzee. This conclusion is in agreement with the hypothesis proposed by Myers et al. (2005) [24] that, at the Mb scale, the regional hotspot density and activity remains fairly constant over relatively long evolutionary time, despite fine-scale changes in hotspot location.

This conclusion (rapid local fluctuation of hotspot location, but conservation of regional hotspot density) may explain the first paradox raised by Spencer and colleagues [23]: although at a given time, hotspots occupy only 3% of the genome, on the long term, a large fraction of the genome may be affected by hotspot activity.

The conservation of recombination rate at the Mb scale probably reflects some constraints on the distribution of crossover events. Indeed it is known that in mammals (as in many other taxa), there is a requirement of one crossover per chromosome arm to ensure a proper segregation of chromosomes during meiosis (for review, see [51]). This constraint leads to a higher crossover rate in smaller chromosome arms [15], [51]–[53].

The resolution of the HAPMAP genetic map allowed us to investigate the correlation between GC* and recombination at finer scale. The strength of correlations decreases with smaller window size (Table 1), and becomes very weak below 200 kb, possibly because at this scale, other factors contribute to variations in substitution patterns. Interestingly, the correlation between GC* measured in human and chimpanzee orthologous regions remains high (R^2^>40%), up to 200 kb (Table 1) (NB: this is an underestimate because the accuracy of the measure of GC* decreases with smaller window size [54]). Moreover, GC* measured in the chimpanzee lineage shows the exactly same correlation to the rate crossover measured in human populations as GC* measured in the human lineage (Table 1). This suggests that the regional hotspot density remains conserved between human and chimp at least up to the 200 kb scale.

### Strong Correlation between Substitution Patterns and Male-Specific Crossover Rates

The rate of meiotic recombination differs between males and females: the rate of crossover in autosomes is on average 65% higher in females than in males, and the genetic maps are poorly correlated between the two sexes (crossover rates in females are higher around the centromeres, whereas those in males tend to be higher towards the telomeres) [45]. In a previous work, we had found that GC* correlated more strongly with female than with male recombination rate [15]. However, this result was based on the analysis of 33 loci only, and the difference became non-significant after excluding only one data point [15]. Moreover, the analysis of substitution patterns in Alu repeats lead to the opposite conclusion [17]. To resolve that issue, we analyzed in our whole-genome data set, the correlation between GC* and sex-specific crossover rates provided by the deCODE genetic map. We found that on autosomes, GC* is much more strongly correlated to male crossover rate (R^2^ = 0.27) than to female crossover rate (R^2^ = 0.15). On the X chromosome, that recombines only in females (we excluded pseudo-autosomal regions from our analyses), we found a correlation between GC* and crossover rate that is weaker than that observed in autosomes (deCODE: R^2^ = 0.22, HAPMAP: R^2^ = 0.17). Thus, we confirm the observation of Websters and colleagues [17], that male crossover rate is a much stronger predictor of GC* than female crossover rate.

### BGC Model: Confronting Predictions with Observations

We have previously reported different observations that support, qualitatively, the BGC model for the evolution of isochores [16]. However, it is important to quantify more precisely the prediction of the BGC model: given that recombination occurs essentially in hotspots that cover only 3% of the genome, that the BGC effect in hotspots is weak, and that hotspots have a short lifespan, is it possible that BGC drive the long term evolution of the base composition of Mb-long sequences? To address that issue, we performed theoretical calculations to quantify the potential impact of BGC on genome evolution.

We considered a model of genome evolution, where sequences are only subject to mutations and to BGC (i.e. no selection). Advancing a model by Lipatov and colleagues [55], we assume here a model in which BGC only occurs in hotspots, with all other DNA undergoing neutral evolution. Let the fraction of the genomic region that is involved in a hotspot be *f*. We assume that the mutation process is the same both in and out of hotspots and that the mutations rate from W→S is *μ_w_*_→*s*_ and the rate from S→W is *μ_s_*_→*w*_. Then the rate of substitution from W→S in a given genomic region is:
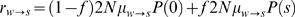
(2)and the rate from S→W is
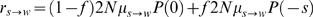
(3)where *N* is the effective population size and *P(s)* is the probability that a mutation subject to BGC of strength *s* will be fixed. BGC behaves just like selection of a semi-dominant mutation [21] so:
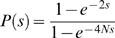
(4)
*P*(0) is the probability that a mutation, which is not subject to BGC, is fixed under random drift: i.e. *P*(0) = 1/2*N*.

The rate of recombination varies along chromosomes, as a consequence of variations in density and intensity of recombination hotspots [24]. Thus, the impact of BGC in a given genomic fragment depends on the local density and intensity of recombination hotspots. We considered genomic fragments of 1 Mb. We assume that at this genomic scale, and for the period of time considered here (i.e. corresponding to the human/chimpanzee divergence), the hotspot density and average intensity remain constant during time. However, we do not assume that hotspots remain at the same position within the fragment. To investigate independently the impact of hotspot density and intensity on genome evolution we considered two models: in the first one (M1), we consider that the rate of recombination in a given genomic fragment varies only through the density in recombination hotspots, which are assumed to have all the same intensity; in the second one (M2), we keep the density of hotspots constant over across the chromosome but vary the intensity of hotspots in the genomic fragments. The distribution of densities (for M1) and intensities (for M2) are chosen to mimic the actually observed genome wide distributions of recombination rates in the human genome.

The BGC coefficient (*s*) depends on the intensity of the hotspot (*i*) (i.e. its rate of recombination), the length of the heteroduplex (*h*) and the bias in the repair of W:S mismatches (*b*). It is known that *i* varies among hotspots [56]. There is presently no evidence for variations of *b* and *h* along chromosomes. Hence we will simply assume here that variations in *s* reflect variations in *i*, so:
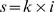
(5)where *i* is the rate of recombination and *k* a constant factor.

We used equations (2) and (3) to compute S→W and W→S substitution rates predicted by the BGC model, independently for transversions, non-CpG transitions and CpG transitions. S→S and W→W substitution rates are not affected by BGC, and hence were assumed to be identical to their mutation rates and constant across the genome.

**Figure 5:**
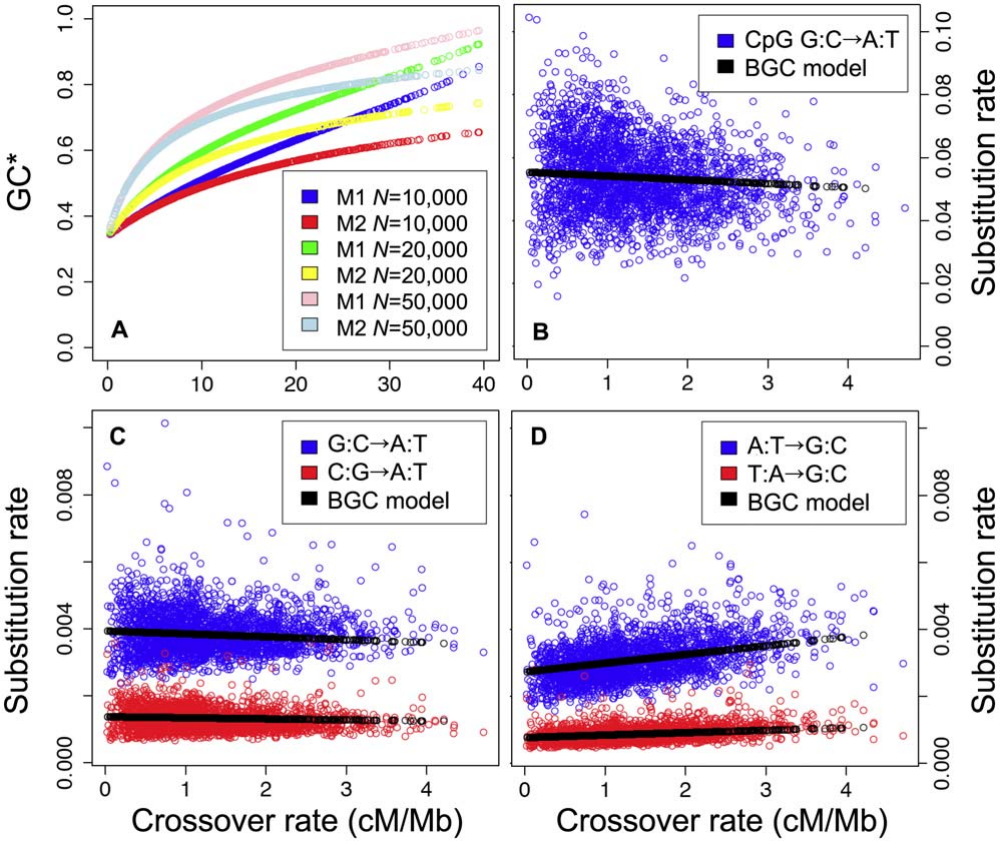
Predictions of the BGC model and comparison with observed data in human autosomes. (A) Predicted GC* *vs.* crossover rate for different parameters of the BGC model (M1 or M2 (see text)) and different effective population sizes (*N*). (B–D) Correlations between base-specific substitution rates and crossover rates in human autosomes (1 Mb windows). Blue and red dots: observed data. Black dots: predictions of the BGC model (Model M1, *N* = 10,000).

For our calculations, we chose parameters as realistic as possible. We considered a sequence with a base composition typical of the human genome (i.e. GC-content = 40.6%, CpG density = 1%) (NB: we do not assume that the base composition of the sequence is at equilibrium). We calculated substitution rates predicted by the model (at CpG and non-CpG sites) for a period of time corresponding to the human/chimpanzee divergence. To estimate mutation rates, we took from our above analyses the average substitution rates measured in fragments of low recombination of human autosomes (<0.44 cM/Mb, i.e. corresponding to the first 10% of the dataset). Recombination rates in 1 Mb-long fragments of human autosomes were taken from HAPMAP data, and range from 0.02 cM/Mb to 4.71 cM/Mb (1.33 cM/Mb on average). Recombination hotspots are typically 2 kb long, and cover 3% of our genome [24]. Thus, the average intensity of recombination hotspots (*i*) is 44.4 cM/Mb. In model M1, we consider that *f* varies from 0.05% to 10.7% (with *i* = 44 cM/Mb), whereas in model M2, *i* varies from 0.66 cM/Mb to 157 cM/Mb (with *f* = 3%). We considered an effective population size *N* = 10^4^. We presently have no direct measure of the BGC parameter within recombination hotspots, but the order of magnitude of this parameter can be estimated from the analyses of Spencer and colleagues [23]. These authors computed the average BGC parameter (4*Ns*) in large genomic regions by fitting a population genetics model to the frequency distribution of SNPs in human populations [23]. They divided their genome-wide data set into quintiles of recombination rate and found that the average BGC parameter increases 2.6 fold from 4*Ns* = 0.5 in genomic regions of low recombination (i.e. the first 20%, average crossover rate = 0.42 cM/Mb) to 4*Ns* = 1.3 in regions of high recombination (i.e. the top 20%, average crossover rate = 2.54 cM/Mb) [23]. Thus, in these highly recombining regions, the average value of *k* is *k_ref_* = 7.25 10^−7^ (see equation (5)). We computed GC* according to the substitution rates predicted by models M1 and M2 for several values of *k* (from *k* = *k_ref_* to *k* = 10 *k_ref_*). The values of *k* for which the correlation between GC* and crossover rate was the closest to the one observed in the data were *k* = 4*k_ref_* and *k* = 5*k_ref_* (i.e. on average, within recombination hotspots, 4*Ns* = 5.2 to 6.5). The hypothesis that *k* might be 4 to 5 times higher in recombination hotspots than in the set of highly recombining regions analyzed by Spencer and colleagues is perfectly plausible, given that the average crossover rate within recombination hotspots is 17 times higher (44.4 cM/Mb). The correlation between GC* predicted by model M1 (with *k* = 4*k_ref_* = 2.9 10^−6^) and the rate of crossover in the human genome is presented in Figure 1b (green dots). The slope of the correlation is very close to that observed in real data (blue dots). Note that for the range of recombination rate observed in the human genome (0.02 cM/Mb to 4.71 cM/Mb), models M1 and M2 give very similar predictions (Figure 5a). Thus, with realistic parameters, the BGC model perfectly predicts the correlation between GC* and crossover rate. Notably, it correctly predicts the erosion of GC-rich isochores: even in regions of high recombination, BGC is not strong enough to maintain a GC-content as high as in present GC-rich isochores. Of course, the correlation is much more noisy in real data than predicted by our model, because 1) our calculations do not include any stochastic effect and 2) in real data, the pattern of mutation is not constant across the genome.

Interestingly, the model predicts that the impact of recombination on S→W and W→S substitution rates in genomic fragments is not symmetric. When BGC is not effective (i.e. *Ns*≪1), substitution rates converge towards mutation rates. But as the strength of BGC increases then *P(s)* converges to 1 and *P(−s)* to 0. Thus, we obtain:
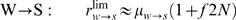
(6)

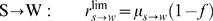
(7)Hence, whereas BGC can strongly increase *r_w_*_→*s*_ (by a factor *f2N*), the decrease in *r_s_*_→*w*_ is limited by *f*. Again, this prediction of the model fits perfectly the observations: whereas W→S substitution rates are positively correlated to crossover rates (Figure 4d), S→W substitution rates show no or weak negative correlations (Figure 4b, 4c). The slopes of the correlations fit very well with the predictions of the BGC model (Figure 5b,c,d). Thus, the BGC model predicts the observed positive correlation between the total substitution rate and recombination (Figure 4a). Note that the BGC model predicts no correlation between recombination rate and W→W or S→S substitution rates. In agreement with this prediction, the rate of A:T→T:A substitutions is not correlated to crossover rate (R^2^ = 0.003, Figure 4c). However, the rate of C:G→G:C substitutions is weakly positively correlated to crossover rate (R^2^ = 0.06, Figure 4d), and the correlation remains significant after controlling for the effect of variations in GC-content (Table 2).

### BGC Model: Substitution Hotspots in Recombination Hotspots

S→W and W→S substitution rates within recombination hotspots are given by the following equations (see above equations 2–4 for the notations):
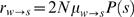
(8)

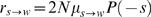
(9)Given the BGC parameters inferred previously for an average recombination hotspot (4*Ns* = 5.2), S→W substitution rates are predicted to be 35 times smaller than their corresponding mutation rates, whereas the W→S substitution rates are predicted to be 5 times higher than their mutation rates. Thus the S→W substitution rates at CpG and non-CpG sites are respectively 11 times and 121 times smaller than W→S substitution rates. Hence, the equilibrium GC is almost 100% within hotspots.

Note however that, for the divergence time considered here, the total substitution rate predicted within recombination hotspot is only two times higher than in the rest of the genome (1.1% vs. 0.5%) (this is because the 5-fold increase in *r_w_*_→*s*_ is compensated in part by the absence of S→W substitutions). Thus, for an average recombination hotspot (i.e. 4*Ns* = 5.2), the impact of BGC on the local substitution rate is relatively modest. Moreover, given that recombination hotspots move rapidly, most of them should not create substitution hotspots.

However, the most highly active recombination hotspots are predicted to result in substitution hotspots. For example, in the human genome, the intensity of the most extreme hotspot is about 450 cM/Mb [56]. The rate of substitution in that hotspot is predicted to be about 11.1%, i.e. about 20 times higher than in the rest of the genome. Thus, the BGC model predicts the existence of substitution hotspots, characterized by a very strong GC-bias. Again, this prediction fits perfectly with the observations: the analysis of substitution hotspots in the human genome revealed that the pattern of substitution in these hotspots is strongly biased towards GC, and that the density in such substitution hotspots is positively correlated to the crossover rate [57].

### BGC Model: The Impact of Recombination Rate and Effective Population Size

Our model predicts that the BGC process is presently too weak to maintain GC-rich isochores in the human genome: GC* in Mb-long regions is predicted to vary in the genome from 34% (in regions of lowest crossover rate) to 42% in regions of highest crossover rate (Figure 1b). However, it is known that recombination rates and effective population sizes vary widely among taxa. To quantify the potential impact of BGC in other species, we computed GC* (in 1 Mb fragments) for higher effective population sizes (up to 50,000) and for higher recombination rates (up to 40 cM/Mb), all other parameters being kept unchanged. As shown in Figure 5a and Table 4, the BGC model predicts the formation of very GC-rich isochores in species with higher effective population sizes or recombination rates.

It should be noted that this range of parameters is realistic. For example, in chicken, the crossover rate ranges from 2.5 cM/Mb in macrochromosomes to 21.1 cM/Mb in microchromosomes [58]. If we consider the other parameters (mutation rates, BGC coefficient, population size) as being the same as in human, this would correspond to a predicted GC* of about 39% in macrochromosomes and 57% (model M2) to 64% (model M1) in microchromosomes. Thus the BGC model predicts a strong isochore structure in chicken.

**Table 4:** Predictions of the BGC model and comparisons with observed values.

		*N*	Whole genome					Low recombination			High recombination		
			Rec. rate	*f*	*i*	GC*	t1/2	Rec rate	GC*	t1/2	Rec. rate	GC*	t1/2
			(cM/Mb)				(Myrs)	(cM/Mb)		(Myrs)	(cM/Mb)		(Myrs)
Model	M1	10000	1.3	3%	44.4	0.37	458	0.3	0.35	469	2.9	0.40	441
	M2	10000	1.3	3%	44.4	0.37	457	0.3	0.35	472	2.8	0.39	429
	M1	10000	13.5	30%	44.4	0.55	362	2.9	0.40	441	29.1	0.71	276
	M2	10000	13.4	3%	444.3	0.51	312	2.9	0.40	428	29.5	0.62	204
	M1	20000	13.4	30%	44.4	0.62	261	3.0	0.44	392	29.3	0.78	156
	M2	20000	13.2	3%	444.3	0.58	239	2.8	0.43	388	29.8	0.71	128
	M1	50000	13.2	30%	44.4	0.73	152	2.8	0.52	306	29.8	0.88	66
	M2	50000	13.2	3%	444.3	0.69	145	2.7	0.51	304	29.1	0.81	62
Observations			1.3	3%	44.4	0.37	470	0.3	0.34	498	2.9	0.40	423

The stationary GC-content (GC^*^) and the half-time of the evolution of GC-content (t_1/2_) predicted by the BGC model are given for different values of the parameters: model M1 or M2 (see text). *N*: effective population size. Rec. rate: genome average recombination rate. *f*: fraction of the genome involved in recombination hotspots. *i*: average intensity of recombination hotspots (cM/Mb). GC^*^ and t_1/2_ are also given for genomic regions of low and high recombination (corresponding respectively to the top 10% of lowest or highest recombination rate in the data set). t_1/2_ is computed assuming that human and chimp diverged 6 Myrs ago.

### BGC Model: Speed of Evolution of GC-Content

Another important parameter to consider is the speed at which the GC-content of a genome can evolve. As an estimator of that speed we can compute the half time of the process (*t*_1/2_) i.e. the time necessary to divide by two the difference between the present GC-content and the equilibrium GC-content.

If *F_GC_* is the frequency of GC nucleotides in the sequence then the change in the frequency of *F_GC_* is:
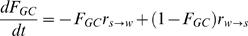
(10)The equilibrium value of *x* can therefore be found by solving the equation 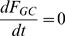


In a simple model of sequence evolution, with constant and uniform substitution patterns along each genomic fragment, *t*_1/2_ can easily be computed:
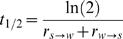
(11)The BGC model predicts that substitution patterns should differ in recombination hotspots compared to the rest of the region. However, if we assume that recombination hotspots move very rapidly relative to *t*_1/2_ and randomly in a given genomic fragment (their density remaining constant), then the long-term patterns of substitution can be considered as uniform and constant. Hence, given equations (2) and (3) we obtain:
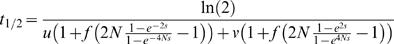
(12)
Table 4 gives the predicted values of *t*_1/2_ for different recombination rates and effective population sizes. In absence of BGC (i.e. no recombination) *t*_1/2_ is about 470 Myrs. In other words, under a standard neutral model, the evolution of GC-content is an extremely slow process. But when BGC is effective, the evolution of GC-content can be much faster (e.g. 62 Myrs in a genomic region of high recombination rate (30 cM/Mb) in a species with large population size (*N* = 50,000)). To estimate *t*_1/2_ more precisely, it would be necessary to take into account the dynamics of movement of recombination hotspots. Presently, little is known about this dynamics, except that the lifespan of recombination hotspots is much shorter than 6 Myrs (the location of hotspots is not conserved between human and chimpanzee). The assumption that hotspots move very rapidly relative to *t*_1/2_ is therefore correct.

Hence, contrarily to the standard neutral mutational model, the BGC model predicts that the evolution of GC-rich isochores can be very rapid in species with large population size and high recombination rate. Thus, the BGC model provides a realistic explanation for the rapid origin of GC-rich isochores in the last common ancestor of amniotes, 310 to 350 Myrs ago [59],[60].

## Discussion

We analyzed the pattern of substitutions that have occurred in the human lineage, since the divergence with chimpanzee. Multivariate regression analyses show that two parameters (the crossover rate and the distance to telomeres, LDT) have a major impact on genome evolution, by affecting the relative proportion of W→S and S→W substitutions. The GC-content of sequences also affects their pattern of substitution (notably at CpG sites). However, the impact of GC-content on the evolution of base composition is relatively weak compared to the two other parameters.

Crossover rate and LDT are two predictors of recombination rate. Taken together, these two variables explain 47% of the variance in GC* at the 1 Mb scale. Thus, our results indicate that recombination is the major determinant of the evolution of GC-content in primates. It should be stressed that the correlation between GC* and the recombination rate is certainly underestimated, because crossover rate and LDT are not expected to be perfect predictors of the average recombination rate in the human lineage during the last 6 Myrs. Note that contrarily to estimates of recombination rates, the measure of GC-content is virtually free of noise. Moreover, given the evolutionary distance considered here, the temporal variations in GC-content are negligible (human and chimpanzee orthologous sequences are 98% identical). Thus, whereas the impact of recombination on substitution patterns is underestimated, the impact of GC-content is not. This reinforces our conclusion that the impact of recombination on sequence evolution is much stronger than the impact of GC-content.

Our results demonstrate that recombination has been driving the evolution of GC-content in the human lineage, at least during the last 6 million years. In chicken chromosomes there is also a strong correlation between crossover rate and GC-content [58]. Thus, it appears that the same process, associated to recombination, is responsible for the evolution of GC-rich isochores in the genomes of mammals and birds. Three different hypotheses can be proposed to explain this effect of recombination: selection, mutation or BGC. We will hereafter discuss in detail each of these models.

### The Biased Gene Conversion Model

Allelic gene conversion, i.e. the copy/paste of one allele onto the other one at heterozygous loci, occurs during meiotic recombination [61]. Different authors have proposed that this process could be biased toward GC, so that an AT/GC heterozygote would produce more GC than AT gametes [11],[14],[19],[20], leading to a higher probability of fixation of GC over AT alleles. This bias in the process of gene conversion should therefore lead to an increase of GC-content in highly recombining regions. It should be noted that there is experimental evidence for a GC-biased Base-Excision Repair process in mammals [20],[62]. Thus, this provides a plausible mechanistic basis to the BGC model.

The BGC process should result in a fixation bias in favor of GC alleles, especially within recombination hotspots. Analyses of polymorphism at silent sites (synonymous codon positions or non-coding sequences) are consistent with these predictions: GC-alleles (i.e. alleles resulting from a W→S mutation) segregate at a higher frequency than AT-alleles in human populations [4],[23],[34],[63] and that this bias is strongest at the center of recombination hotspots [23],[64].

We show here that the observed relationship between GC* and recombination rate fit very well with the predictions of the BGC model, using realistic parameters (Figure 1b). Interestingly, our modeling shows that recombination should have a strong impact on the rate of W→S substitution, but only a weak effect on S→W substitutions. Again, this prediction of the BGC model fits precisely with the observations (Table 2, Figure 5). Interestingly, this model also predicts the observed positive correlation between the total substitution rate and recombination (Figure 4a).

As mentioned in the introduction, Spencer and colleagues [23] pointed out several issues with the BGC model. Notably they argue that in humans, the population-scaled BGC coefficient is too weak for BGC to have a strong effect on base composition evolution. Hence they conclude that BGC is not sufficient to account for the origin of GC-rich isochores. We agree on the first point: our calculations show that, given the density in recombination hotspots in the human genome and the estimated effective population size in our species, BGC is not efficient enough to maintain the base composition of GC-rich isochores. And in fact this prediction fits perfectly with the observations: the analysis of substitution patterns indicate that there is an erosion of the isochore structure of our genome (Figure 1a) [15], [16], [33]–[38]. However, the fact that BGC is presently weak in the human species does not exclude that BGC might have been more active in the past and might still be efficient in other species. Indeed, our calculations show that in species with an effective population size as large as humans but with a rate of recombination as high as chicken, BGC can lead to a strong isochore structure. Interestingly, it has been noticed that, contrarily to primates where GC-rich isochores are being eroded, the genomic heterogeneity in GC content along the chicken lineage is increasing [65].

### Mutagenic Effect of Recombination?

An alternative hypothesis to explain the observed variations in GC* is that recombination could affect the pattern of mutation. There is evidence, based on direct experiments in yeast, that recombination can be mutagenic [66], and it has been speculated that this might also be the case in mammals [67]–[71]. Thus if recombination promotes W→S mutations, this could explain the correlation between GC* and recombination.

There are two problems with this model. First, there is *a priori* no reason why recombination should affect more strongly W→S mutation rates than other mutations. Second, this mutational model does not fit with the frequency spectrum of polymorphism at silent sites. In fact, under the hypothesis that recombination promotes W→S mutations, in a recent recombination hotspot, one would expect an excess of recent GC-alleles. Thus, on average, GC-alleles should segregate at a lower frequency than AT-alleles. In more ancient recombination hotspots the frequency spectra is expected to be the same for GC and AT alleles. Thus, the fact that GC-alleles segregate at higher frequency than AT-alleles and that this bias is stronger within recombination hotspots [23],[64] is opposite to the pattern expected if recombination promoted W→S mutations.

It has been recently shown that the apparent difference in frequency spectrum between GC and AT alleles was partly due to an artifact of parsimony, resulting form the fact that S→W substitution rates are generally higher than W→S substitution rates [72]. Such an artifact however cannot account for the observation that the excess of GC-alleles at high frequency increases within recombination hotspots (in fact, since recombination promotes W→S substitutions, this parsimony artifact should induce the opposite pattern, i.e. an excess of AT-alleles segregating at high frequency within recombination hotspots). Thus, the higher frequency of GC-alleles within recombination hotspots is a clear demonstration that recombination induces a fixation bias, favoring GC-alleles. Hence, this rules out the hypothesis that the correlation between GC* and recombination is a mere consequence of mutagenic effects of recombination.

This does not demonstrate however that the impact of recombination on sequence evolution is exclusively due to the BGC process. Indeed, as shown previously, the BGC model predicts that recombination should have a weak negative effect on S→W substitution rates and no effect on S→S and W→W substitution rates. In contradiction with those predictions, partial correlation analyses indicate that, after controlling for GC-content, all base-specific substitution rates tend to be positively to recombination rate (Table 2). This positive effect of recombination on S→W, S→S and W→W substitution rates is weak but significant (the strongest effect is observed for S→S substitution, R^2^ = 0.06, Figure 4d). One possible explanation is that, besides its effect on fixation probability *via* the BGC process, recombination might also be mutagenic [67].

However, given the weakness of these correlations, we cannot exclude that it results from indirect relationships between recombination rate and other parameters. Notably, it has been shown that the divergence time between human and chimpanzee orthologous loci is not constant along chromosomes, because of variations in coalescence times [73]–[75]. Recombination decreases the genetic linkage between sites under selective pressure and flanking neutral sites. Hence recombination is expected to increase coalescence time at neutral sites [76],[77]. Thus, this process could contribute to these positive correlations between substitution rates and recombination rate. In other words, the weak positive correlation between substitution rates and recombination rate cannot be considered as an evidence for a mutagenic effect of recombination.

### Strong Evidence against Selectionist Models of Isochore Evolution

Several authors have proposed that GC-rich isochores might result from selection [3], [5]–[8]. It should be noted that the evolution of isochores affects all kinds of sequences: exons, introns, intergenic regions, pseudogenes, transposable elements [1]. Thus, if selection is at work, this is not on the information content of genomic sequences, but simply on their GC-content. Any selective model should be able to account for the fixation bias observed on SNPs. In other words, these selective models must assume that there is a significant fitness difference between two individuals differing only by a few point mutations in Mb-long isochores. Even the proponents of selective models admit that the change in GC-content resulting from a point mutation is certainly to weak to be detected by selection [8]. Bernardi (2007) recently proposed a ‘neoselectionist theory’ to explain the evolution of isochores [8] but, without any mathematical formulation, this model remains speculative.

A strong argument against these selective models is that they do not predict the observed strong relationship between GC* and recombination. In fact, selective models might predict a weak indirect relationship between GC* and crossover rate. Indeed, selection is expected to be less efficient in regions of the genome where the rate of crossover is low, because of the so-called Hill-Robertson interference (reviewed in [78]). Thus, if there is a selective pressure in favor of a high GC-content, then this Hill-Robertson interference would predict a positive correlation between GC* and the rate of cross-over. The impact of Hill-Robertson interference on selection efficiency is however very weak and affects almost exclusively region where the recombination rate is null [79]–[82]. Hence, it seems very unlikely that this Hill-Robertson interference could explain the strong linear correlation observed between GC* and crossover rate (Figure 1b). Moreover the Hill-Robertson interference depends on the total rate of crossover in populations across generations, occurring both in females and in males. Thus, *a priori*, the correlation between GC* and crossover rate should be the same in males and females. In fact, given that the female effective population size tend to be larger than male effective population size [83] one should expect, if anything, a stronger correlation of GC* with female than male crossover rate. The fact that GC* correlates much more strongly with male than with females crossover rate therefore definitively rules out these selective models.

### Impact of GC-Content on Substitution Patterns: Mutagenic Effect of DNA-Melting?

Fryxell and Zuckerkandl (2000) [13] have recently proposed that isochores might result from a positive feedback loop of sequence composition on substitution patterns: the rate of C→T mutations (notably at CpG sites) depends on DNA melting which in turn depends on GC-content. Thus the rate of S→W mutation is expected to be higher in AT-rich than in GC-rich regions, which should tend to increase the contrast in GC-content between GC-rich and GC-poor isochores [13]. If this process was the main determinant of the evolution of isochores, then we would expect a strong correlation between GC* and GC. Thus, our observation that GC* is much more strongly correlated to recombination rate than to GC, rules out the model of Fryxell and Zuckerkandl as the main explanation for the evolution of base composition.

However, our analyses indicate that, after controlling for recombination rate, the GC-content does have a significant impact on substitution rate. Notably, S→W and S→S substitution rates are negatively correlated to GC-content (Table 2). This observation is consistent with the hypothesis that the rate of cytosine mutation depends on DNA melting [13]. The CpG methylation deamination process shows the strongest dependency on the GC-content. Its overall frequency varies by a factor of two from about 0.07 substitutions per site in GC-poor regions to about 0.035 in GC-rich regions (R^2^ = 0.42, Figure 3b). Although the effect is weaker, W→S and W→W substitution rates are also negatively correlated to GC-content (after controlling for recombination rate, Table 2). This suggests that DNA melting might affect all mutation rates. Thus, the pattern of substitution at a given locus is affected not only by its recombination rate, but also by its GC-content.

### The Impact of Recombination on Substitution Patterns: Crossover and Non-Crossover Events

One of the reasons why HAPMAP and deCODE genetic maps do not provide perfect estimators of recombination rate is that crossovers constitute only a fraction of all recombination events. Indeed, meiotic recombination is initiated by double-stranded breaks, the repair of which leads to the formation of a Holliday junction. This junction is then resolved, either with the exchange of flanking markers (crossover) or without exchange (non-crossover). Both cases involve gene conversion (i.e. non-reciprocal exchange of DNA material between the two chromosomes). Thus, the total rate of recombination (*r*) is given by:
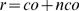
(13)where *co* is the rate of crossover and *nco* the rate of non-crossover. If we call *g* the ratio of non-crossover to crossover, this gives
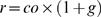
(14)It has been shown that *g* varies along human chromosomes, with some recombination hotspots showing more crossovers than non-crossover and vice versa [84]. The BGC process depends on the total recombination rate (crossover+non-crossover). Thus, GC* is not expected to be perfectly correlated to the rate of crossover.

The analysis of polymorphism in *Drosophila melanogaster* subtelomeric regions indicates that these regions are subject to a high rate of recombination despite a low rate of crossover [85]. Interestingly, our partial correlation analyses show that GC* is negatively correlated to LDT, after controlling for other factors (crossover rate, GC) (Table 2). In other words, near human telomeres, GC* is higher than predicted by crossover rate. This suggests that in mammals, as in drosophila, *g* might increase as the distance to telomere decreases. However, more direct estimates of the total recombination rate will be necessary to validate this hypothesis.

### Male Driven BGC?

We found that GC* is much more strongly correlated with male than with female crossover rates. This confirms previous results based on the analysis of substitution patterns in Alu repeats [17] and in substitution hotspots [57]. A first possible explanation for this observation is that BGC might be stronger in males than in females (male-driven BGC). Given that LDT is much more strongly correlated to male than to female crossover rates (respectively R^2^ = 0.38 and R^2^ = 0.04, at the 1 Mb, in human autosomes), this could explain why LDT is a good predictor of GC*. The strength of BGC depends on three parameters: the length of heteroduplex, the bias in the repair of W:S mismatches and the total recombination rate (crossovers+non-crossovers). For the first two parameters, we presently have no information about possible sex-specific differences. The rate of crossover (in autosomes) is on average 65% higher in females than in males [45]. Thus, BGC is *a priori* expected to be weaker in males than in females. However, the average of the ratio of non-crossover to crossover (*g*) might be different in the two sexes. Thus, it possible that the total recombination rate (and hence BGC) is higher in males than in females.

An alternative explanation is that the strength of BGC is the same in both sexes but that in females the rate of crossover is only weakly correlated to the total recombination rate. Indeed, the ratio of non-crossover to crossover (*g*) varies along chromosomes, and hence the rate of crossover is not a perfect estimator of the total recombination rate. Thus, the lower correlation observed with female crossover rates might simply be a consequence of stronger variations of *g* along chromosomes during female meiosis.

It should be noted that the effective population size of the X chromosome is only ¾ of that of autosomes. Moreover, the level of heterozygosity on the X chromosome is 39% lower than in autosomes (owing to lower mutation rate and reduced effective population size) [86]. Thus, all else being equal, one should expect a weaker impact of BGC on the X chromosome compared to autosomes. This could contribute to the fact that the correlation between GC* and crossover rate is lower on the X than on autosomes.

### Conclusion

Both empirical data and theoretical calculations support the hypothesis that BGC has a strong impact on the evolution of GC-content in amniotes. In fact the BGC model explains most of the properties of isochores and their timing of evolution. Furthermore our results allowed us to reject the alternative models for the evolution of isochores (mutation or selection). Thus, we conclude BGC is the process at the origin of evolution of isochores.

It should be noted that the process that created isochores affected not only silent sites but also coding regions. Indeed, the amino-acid composition of proteins is correlated to the GC-content of the genomic region where the gene is located [87]. In fact, the impact of BGC on substitution patterns can be very strong, even in regions that are under selective pressure (coding sites or regulatory elements). In some cases, BGC overcomes purifying selection and leads to the fixation of deleterious AT→GC mutations [22]. We argue that along with mutation, selection and drift, BGC might be one of the major factors driving genome evolution and that it is essential to take this process into account if we want to be able to interpret sequences.

Finally we note that GC* provides information about the long-term total recombination rate (crossovers+non-crossovers). Notably, our results indicate that at the 1 Mb scale, recombination rates are conserved between human and chimpanzee. Thus, the analysis of recent substitution patterns can provide an insight into the evolution of recombination and the distribution of crossover and non-crossover events along chromosomes.

## Material and Methods

### Genomic Data

We analyzed genome-wide multiple sequence alignments (multiz alignments) for the three species *Homo sapiens* (assembly hg17), *Pan troglodytes* (panTro1), and *Macaca mulatta* (rheMac1), which have been downloaded from the UCSC Genome Browser website. A total of about 2350 Mb of human sequence segments are aligned to chimp and macaque segments. To ensure a high quality of the multiple alignment we include in our analysis only those sequence segments that are located on human autosomes or X chromosome, are at least 1500 bp long, and have less than 10% positions involving a gap in one of the three species. Further, we remove from the aligned sequences those segments that overlap with coding segments (exons) according to the annotation of human genome taken from the Ensembl project [88]. This way we are left with alignments of non-coding sequences from human, chimp, and macaque covering about 1 Gb of the human genome.

For our analysis we partition each human chromosome in non-overlapping windows of constant length. We used the following window lengths: 100 kb, 200 kb, 500 kb, 1 Mb, 2 Mb, 5 Mb, and 10 Mb. For each of those tilings and in each of its windows we collected all triple alignment segments falling into a window and used them to estimate the substitution frequencies as described below. Depending on the window length we measure substitution frequencies in 320 (for the 10 Mb) to 30,400 (for the 100 kb) windows along the human genome. For some analysis we further restricted the alignments to intergenic or intronic sequences. The additional masking of simple sequence repeats (that make up only a small fraction of the genomic DNA) does not change the estimates of substitution frequencies or the stationary GC content (not shown).

Data about crossover rates in chromosomal regions has been obtained from the HAPMAP project [44] and from the deCODE genetic map [45]. The crossover rates for the sequence windows were computed as a weighted average of crossover rates in chromosomal regions that overlap with the corresponding window.

### Model of Nucleotide Substitution

The nucleotide distribution of most contemporary genomes is still evolving. Whereas the present time GC-content can easily be measured from the genomic sequence, a more careful analysis is necessary to estimate the future stationary GC-content. Our approach to this problem is to measure the nucleotide substitution frequencies from multiple alignments and to extrapolate from them the stationary GC-content. However, the measurement process must not assume neither the stationarity of the nucleotide distribution nor the time reversibility of the nucleotide substitution process. These two assumptions are often made during a phylogenetic analysis and therefore we introduce a new methodology which does not make these assumptions and which gives us more power to interpret our results.

We distinguish two classes of nucleotide substitution processes. First, there are the 12 distinct substitution processes of independently evolving nucleotides. The rates of all these processes, α→β, will be denoted *r*_α→β_, where Greek letters represent nucleotides A, C, G, or T. These rates measure the number of substitutions per base pair and per time in a sufficiently small time interval such that multiple substitutions at the same position can be disregarded. For convenience we write those rates into a 4×4 matrix with off-diagonal matrix elements 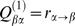:
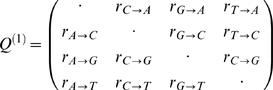
(15)The diagonal elements are constrained by the condition that every column adds up to zero, i.e. 
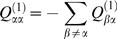
. In this article we consider the general reverse complement symmetric substitution model, which accounts for the fact that a nucleotide substitution on one strand of the DNA is accompanied by a nucleotide substitution on the reverse strand to ensure the correct Watson-Crick base pairing before and after the mutation process. This is incorporated into our model by having only 6 free parameters *r_AT_*_→*TA*_: = *r_A_*_→*T*_ = *r_T_*_→*A*_, *r_GG_*_→*GC*_: = *r_C_*_→*G*_ = *r_G_*_→*C*_, *r_AT_*_→*CG*_: = *r_A_*_→*C*_ = *r_T_*_→*G*_, *r_CG_*_→*AT*_: = *r_C_*_→*A*_ = *r_G_*_→*T*_, *r_AT_*_→*GC*_: = *r_A_*_→*G*_ = *r_T_*_→*C*_, *r_GC_*_→*AT*_: = *r_G_*_→*A*_ = *r_C_*_→*T*_) assuming the equality of complementary nucleotide substitutions. In the above notation the time evolution for the probability, *P*_β_(*t*), to find a nucleotide β at time *t* is given by the Master equation
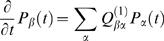
(16)The second class of substitution processes we want to consider are those that depend on identity of the neighboring nucleotide. One such process is the CpG methylation deamination process that triggers the substitution of cytosine in CpG resulting in TpG or CpA. It is of particular importance to include this process in models for nucleotide substitutions in vertebrates, since this process is in fact the predominant substitution process for them [35]. To include this process we have to consider the dynamics of three nucleotides, which is governed by a 64x64 rate matrix
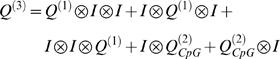
(17)where *I* is the 4×4 identity matrix. The first three terms in the above expression represent the neighbor independent nucleotide substitutions on the three sites (modeled using the matrix *Q* ^(1)^). The last two terms in the above expression represent additional neighbor dependent contributions to the dynamics. For the CpG process, the 16x16 matrix 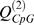 is given by
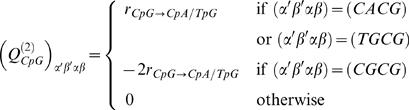
(18)It encodes the modeling of the transition from CpG to CpA or TpG with rate *r_CpG_*_→*CpA/TpG*_. Please note that in principle it is possible to include more than just this one neighbor dependent process. Rows and columns of *Q* ^(3)^ are now labeled by triplets of nucleotides β_1_β_2_β_3_ and α_1_α_2_α_3_. The explicit form of the matrix *Q* ^(3)^ is given in the Supplementary Text S2. As above, the time evolution for the probability, 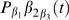, to find three consecutive nucleotides β_1_β_2_β_3_ is given by a Master equation
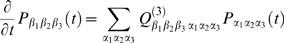
(19)This differential equation for the vector of probabilities 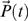 can be solved by matrix exponentiation
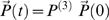
(20)where 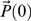 is the initial condition and the 64×64 matrix
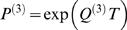
(21)encodes the probabilities of (potentially multiple) substitutions from a triplet α_1_α_2_α_3_ to β_1_β_2_β_3_ in a finite time interval *T*. This probability is given by the matrix element
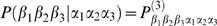
(22)Without loss of generality, we will choose *T* = 1 in the following. With this choice the rates 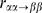 and *r_CpG_*_→*CpA*/*TpG*_ are equal to the nucleotide substitution frequencies. The above expression will be used below to compute the likelihood of nucleotide substitutions along the branches in a given phylogeny. Once the nucleotide frequencies are known it is easy to compute the stationary single- and di-nucleotide frequencies considering the *T*→∞ limit of the above solution by raising *P* ^(3)^ to a high power.

### Maximum Likelihood Framework

Let us consider a *N* species, which are annotated to the leaf nodes *j* = 1,…,*N* of given phylogeny (see Supplementary Figure S1 for an example). In addition to the leaf nodes there are *M* internal nodes at the various branch points *j* = *N*+1,…,*N*+*M* in the phylogeny. The number of internal nodes *M* is always smaller than the number of leaves; it is maximal if the phylogeny is bifurcating in which case we have *M* = *N*−2. Let us further root the phylogeny at the root node *j* = 0. All branches in the phylogeny can now be denoted by ordered pairs (*i*, *j*) with *i*≠*j* and where we assume that the node *i* is always nearer to the root than the node *j*.

For the species on the leaf nodes *i* = 1,…,*N* we have nucleotide sequences 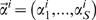 of length *S*. These sequences are assumed to be correctly aligned, i.e. homologous sites 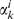 have the same positional index *k* = 1,…,*S*. If gaps are present in the alignment we exclude those sites from further analysis.

The likelihood to observe the current day sequences on the leaf nodes under a given phylogeny is
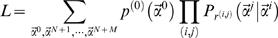
(23)where 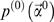 denotes the probability to have 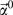 as the ancestral sequence, which need not to be the stationary distribution of any of the used nucleotide substitution models, and 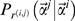 are the transition probabilities of sequences along the edge (*i*, *j*) (parameterized by a sets of substitution rates *r* ^(*i*,*j*)^). The summations in this expression have to be taken over all 4*^S^* configurations of sequences at the root node *j* = 0 and internal nodes *j* = *N*+1,…,*N*+*M*. Note that the substitution models along each branch are assumed to be time homogeneous, i.e. their substitution rates do not change along a single branch. However, these substitution rates might change from one branch to another and we do not put any constrains on the rates along time consecutive branches, e.g. (*i*, *j*) and (*j*, *k*), or on the rates along branches originating from a single node, e.g.(*i*, *j*) and (*i*, *k*). As found in the main text, substitution frequencies depend on the GC content. However it is not necessary to include this effect explicitly into our model, since the GC content only evolves very slowly and this effect can very well be disregarded on the time scales of our study.

### Maximizing the Likelihood Function – Estimation of Substitution Frequencies

The likelihood function introduced above can be used to estimate substitution frequencies from multiple alignments of nucleotide sequences from contemporary species. For a given alignment, the likelihood function can be maximized by varying all parameters *r* ^(*i*,*j*)^ attached to each branch and the ancestral nucleotide distribution. This yields maximum likelihood estimators of the substitution frequencies.

#### The Neighbor Independent Case

The likelihood function introduced above simplifies drastically if nucleotides evolve independent from each other. The likelihood factorizes and can be written as
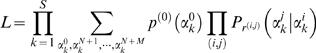
(24)where *p* ^(0)^(α) is now the nucleotide distribution at the root node and the substitution probabilities 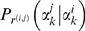 can be calculated using formulas in the previous section. An equivalent expression for the likelihood was already given by [32].

Please note that the computation of the likelihood by ‘pruning’ [32] is possible and allows the effective summation over all configurations of internal nodes. However the ‘pulley principle’ [32] cannot be applied since we consider also irreversible substitution models along the edges of the phylogeny which do not need to obey the detailed balance condition. In general, the likelihood has to be maximized over the (*N*+*M*)×6 free substitution frequencies (for the models along the *N*+*M* branches in the phylogeny) and the ancestral nucleotide frequencies *P* ^(0)^(α) (3 additional free parameters). The maximization can easily be achieved using Powell's algorithm [89], which outperforms other algorithm that make explicit reference also to partial derivatives of the likelihood function.

If the root node *j* = 0 is connected only to two other nodes, say *j*_1_ and *j*_2_, not all of these (*N*+*M*)×6+3 parameters can be fixed by maximizing the likelihood. In this case, the substitution frequencies 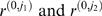 as well as the ancestral nucleotide frequencies *p* ^(0)^(α) have additional degrees of freedom, reflecting the fact that the position of the root node cannot be fixed along the two edges (0, *j*_1_) and (0, *j*_2_) [90]. It can be shown that substitution frequencies along all the other edges, which are not connected to the root, are invariant under this ambiguity and identifiable. If, however, the root is connected to more than two nodes all of the (*N*+*M*)×6+3 frequencies can be fixed.

Using our approach we are also able to reconstruct the nucleotide composition *p* ^(*n*)^(α) at internal nodes *n*, which can be written as:
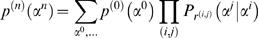
(25)where the product includes only those branches (*i*, *j*) that constitute the path connecting the root node 0 with the internal node *n* in the phylogenetic tree. Likewise, the sum has to be taken over all states of the internal nodes along this path. Note that the possible ambiguities in positioning of the root node do not have an effect on the nucleotide distributions at internal nodes (excluding the root), because ambiguities in the substitution frequencies, 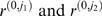, and the ancestral nucleotide frequencies, *p* ^(0)^(α), cancel computing the nucleotide distribution at internal nodes (excluding the root).

#### The Neighbor Dependent Case – Monte-Carlo Maximum-Likelihood Method

Unfortunately, the likelihood in equation (23) does not factorize in the presence of neighbor dependent substitution processes like the CpG methylation deamination process. To still be able to maximize the likelihood we introduce a Monte-Carlo Maximum-Likelihood (MCML) approach, which combines elements of the two methods in a very efficient way. In an iterative fashion we will first (M-step) estimate substitution frequencies for a given ancestral sequence at the internal nodes (using a maximum likelihood approach) and then (E-step) get a new estimate for the sequence at internal nodes for given substitution frequencies (using a Monte Carlo approach).

The iteration is initialized setting the sequences at the internal nodes to be the consensus of all its descendant sequences. If nucleotides at one position are not equal in all descendant sequences one of them is chosen at random. Initializing with a random sequence prolongs but not prevents the convergence of the algorithm to the maximum.

In the M-step substitution frequencies (including the ones of neighbor dependent processes) are estimated from comparisons of ancestral and daughter sequences as described in [35],[54]. This is done using a maximum likelihood approach, which accounts for multiple and back substitutions at the same site, and estimates very accurately the substitution frequencies. The estimation of substitution frequencies is done independently along all edges (*i*, *j*) in the phylogeny yielding sets of frequencies *r* ^(*i*,*j*)^.

In the E-step we update the ancestral sequences at the internal nodes. To do this we make use of a Monte Carlo procedure. Sequentially, we consider the sequences 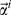 at each internal node *i* = *N*+1,…,*N*+*M* (starting from nodes nearest to the leaves and ascending upwards towards the root).

For each position *k* = 1,…,*S* we propose to update and exchange the nucleotide 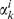 by another nucleotide 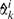. The newly proposed nucleotide is accepted with a certain probability, which is computed using local likelihoods (at position *k* on node *i*):
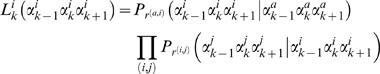
(26)where *a* is the unique ancestral node to the node *i* and the product includes only those branches (*i*, *j*) which originate from node *i* and connect to its descendant nodes *j*. The transition probabilities are defined in equation (21) with substitution frequencies taken from the estimates in M-step. The update 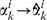 is always accepted if the likelihood increases, i.e. if the likelihood ratio
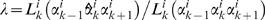
(27)is larger than one. If this ratio is smaller than one the update is only accepted with probability λ. If there is an alignment gap in any of the sequences at position *k*, this position is excluded from our analysis. If there is an alignment gap at one of the neighboring positions, *k*−1 and *k*+1, the nucleotide on position *k* is assumed to evolve only according to those processes that do not include this neighboring site. In principle, this procedure could give rise to misleading estimates when the number of gapped positions in our alignment is high. However, this is not the case in our analysis of closely related genome sequences.

For the root node *i* = 0 we have to modify this update procedure and consider the following local likelihood
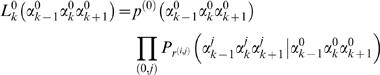
(28)where *p* ^(0)^(α_1_α_2_α_3_) is the trinucleotide distribution of the ancestral sequence 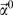, which is assumed to be homogeneous along the sequence and can be measured from the sequence 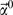 right before starting with E-step. In the above expression we only take the left and right neighbors of the position *k* into account. This approximation to the full likelihood function is very well justified for the CpG methylation deamination process (see in [54] for details and the Supplementary Text S3 for numerical confirmation).

This iteration of the E and M step has to be performed several times until convergence of the substitution frequencies and the tri-nucleotide distribution at the root node is established. In our application this was generally accomplished after about 40 iterations.

As mentioned above for the neighbor independent case, the substitution frequencies of edges connected to the root and the trinucleotide distribution of the ancestral sequence 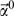 cannot be reconstructed if the root is connected to only two other nodes. This fact however does not influence the convergence of the algorithm. Only the estimates of the substitution frequencies along the two branches connected to the root are generally not correct.

Note that Hwang and Green proposed a method, based on a Bayesian approach, to compute substitution rates, taking into account non-stationary, non-reversible and neighbor dependent substitutions processes [91]. Although the method is different, the principle of their approach is very similar to ours and the results are expected to be the same. The main difference is that Hwang and Green consider all neighbor dependent processes (*WXY*→·*Z*·) and try to estimate the rates for all of them, while we only include the CpG process. Therefore our model has much less parameters and needs much less data (and computation time) to get estimates of these parameters. Note that we showed previously that the inclusion of more neighbor dependent processes is likely not to be significant enhancement of the model [54]. We also reassessed this issue in the current setting without finding significant changes in the estimates of the stationary GC content.

The estimates of substitution frequencies from finite amounts of sequence data are always subject to statistical noise. In general, the uncertainty Δ*r* in the substitution frequencies is proportional to 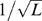, where *L* denotes the sequence length. This prevents us from estimating substitution frequencies from too little sequence data and we therefore do not consider tilings of the human genome smaller than 100 kb.

We performed extensive simulation experiments to test the MCML algorithm, under many different evolutionary scenarios (including the one corresponding to our alignment data set). These simulations showed that, the estimates obtained by our method are very accurate apart for the two branches connected to the root node, for which ambiguities in the positioning of root node prevent reliable estimates of substitution frequencies (for details see Supplementary Materials: Text S3, Figure S1, Figure S2, Table S1, and Table S2).

## Supporting Information

Figure S1 A test phylogeny with 5 leaves which has been used for the first test of the MCML algorithm.
(0.05 MB PDF)

Figure S2 A test phylogeny with 3 leaves (reflecting the situation of human, chimp, and macaque alignments), used for the second test of the MCML algorithm.
(0.04 MB PDF)

Table S1 Nucleotide and substitution frequencies for synthetic sequence data on the phylogeny in Figure S1.
(0.13 MB PDF)

Table S2 Nucleotide and substitution frequencies for synthetic sequence data on the phylogeny in Figure S2.
(0.12 MB PDF)

Text S1 Correlations between the stationary GC-content and the current GC-content, the crossover rate and distance to telomeres.
(0.05 MB PDF)

Text S2 Explicit definition of the matrix Q(3) defined in the Materials and Methods section.
(0.18 MB PDF)

Text S3 Tests of the MCML algorithm using synthetic sequence data.
(0.10 MB PDF)
