# Supplementary material for: The Impact of Recombination on Nucleotide Substitutions in the Human Genome
Source: PLoS Genet. 2008 May 9;4(5):e1000071. doi: 10.1371/journal.pgen.1000071 (PMC2346554; doi:10.1371/journal.pgen.1000071)
Supplement: Figure S1 — A test phylogeny with 5 leaves which has been used for the first test of the MCML algorithm. (0.05 MB PDF) [file pgen.1000071.s001.pdf]

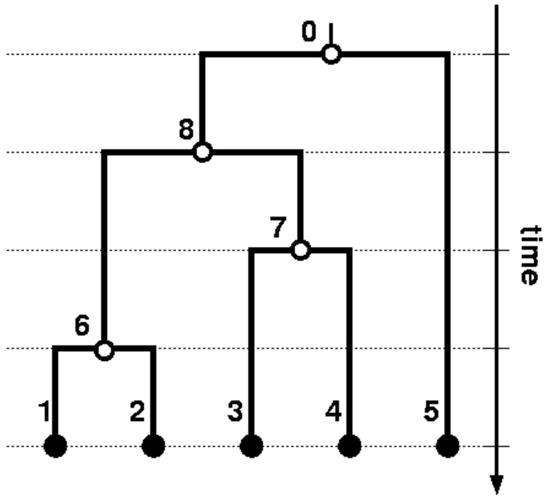

Supplementary Figure S1: A test phylogeny with 5 leaves which has been used for the first test of the MCML algorithm. The branch length is proportional to the transversions frequencies, which have been used to generate synthetic sequence alignments (see also Table S1).
