# Supplementary material for: The Impact of Recombination on Nucleotide Substitutions in the Human Genome
Source: PLoS Genet. 2008 May 9;4(5):e1000071. doi: 10.1371/journal.pgen.1000071 (PMC2346554; doi:10.1371/journal.pgen.1000071)
Supplement: Figure S2 — A test phylogeny with 3 leaves (reflecting the situation of human, chimp, and macaque alignments), used for the second test of the MCML algorithm. (0.04 MB PDF) [file pgen.1000071.s002.pdf]

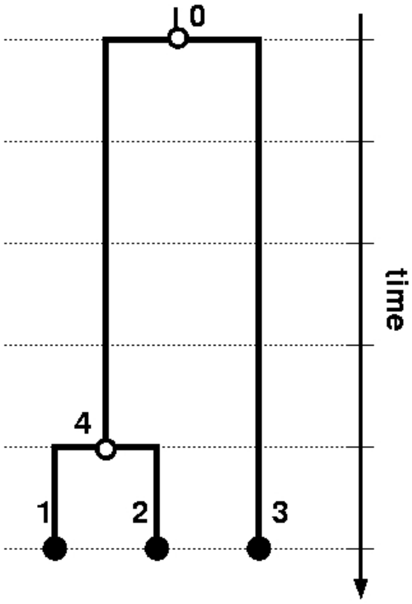

Supplementary Figure S2: A test phylogeny with 3 leaves reflecting the situation of human, chimp, and macaque alignments. For the used substitution frequencies see Table S2.
