# Supplementary material for: The Impact of Recombination on Nucleotide Substitutions in the Human Genome
Source: PLoS Genet. 2008 May 9;4(5):e1000071. doi: 10.1371/journal.pgen.1000071 (PMC2346554; doi:10.1371/journal.pgen.1000071)
Supplement: Table S1 — Nucleotide and substitution frequencies for synthetic sequence data on the phylogeny in Figure S1. (0.13 MB PDF) [file pgen.1000071.s003.pdf]

branch		A to C	A to T	C to G	C to A	A to G	C to T	CG to CA	GC*	GC content
(6,1)	used	0.00100	0.00100	0.00100	0.00100	0.00300	0.00500	0.02000	0.35484	0.48708(47)
	estimated	0.000996(46)	0.001001(45)	0.001002(45)	0.001002(47)	0.003005(81)	0.00501(12)	0.01993(50)	0.3547(61)	-
(6,2)	used	0.00100	0.00100	0.00100	0.00100	0.00500	0.00300	0.02000	0.50735	0.48907(47)
	estimated	0.000997(46)	0.001001(46)	0.001000(45)	0.001000(48)	0.00501(11)	0.002996(91)	0.01996(48)	0.5080(60)	-
(8,6)	used	0.00200	0.00200	0.00200	0.00200	0.00600	0.01000	0.04000	0.35484	0.49013(47)
	estimated	0.001999(92)	0.002004(65)	0.002005(69)	0.00201(10)	0.00610(27)	0.00997(39)	0.03991(84)	0.358(11)	0.49012(47)
(7,3)	used	0.00200	0.00200	0.00200	0.00200	0.00800	0.00800	0.04000	0.43269	0.49096(47)
	estimated	0.001999(70)	0.002002(61)	0.002002(70)	0.002003(72)	0.00803(16)	0.00799(20)	0.03995(72)	0.4334(56)	-
(7,4)	used	0.00200	0.00200	0.00200	0.00200	0.01000	0.00600	0.04000	0.50735	0.49292(47)
	estimated	0.001997(70)	0.002003(62)	0.001996(72)	0.002005(69)	0.01003(17)	0.00598(16)	0.03995(76)	0.5081(51)	-
(8,7)	used	0.00100	0.00100	0.00100	0.00100	0.00500	0.00300	0.02000	0.50735	0.49527(48)
	estimated	0.001003(65)	0.001012(62)	0.001015(67)	0.001015(83)	0.00513(34)	0.00300(22)	0.01996(61)	0.511(14)	0.49525(49)
(0,8)	used	0.00100	0.00100	0.00100	0.00100	0.00300	0.00500	0.02000	0.35484	0.49658(48)
	estimated	0.0019(16)	0.0023(18)	0.0019(16)	0.0022(17)	0.0096(45)	0.0072(74)	0.0200(12)	0.51(16)	0.49649(55)
(0,5)	used	0.00400	0.00400	0.00400	0.00400	0.02000	0.01200	0.08000	0.50735	0.49480(49)
	estimated	0.0028(17)	0.0028(18)	0.0031(17)	0.0032(16)	0.0181(78)	0.0057(46)	0.0791(14)	0.56(13)	-
root node	used	-	-	-	-	-	-	-	-	0.50001(49)
	estimated	-	-	-	-	-	-	-	-	0.4978(42)

Supplementary Table S1: Nucleotide and substitution frequencies for synthetic sequence data on the phylogeny in Figure S1. For each branch (i, j) we report the used and estimated substitution frequencies, the stationary GC content, GC*, and the GC content at the descendant node j. For leaf nodes $j = 1,\dots,5$ the used and estimated values coincide and only one is given. We generated synthetic alignments of 1 Mbp length and report the mean values and standard deviations from 500 independently repeated experiments. The standard deviations in the last digits are given in brackets, e.g. $0.001001(45) = 0.001001 \pm 0.000045$. The substitution frequencies on the two edges connected to the root, (0,5) and (0,8), as well as the GC content at the root node (gray shaded areas) cannot reliably be reconstructed, as mentioned in the text.
