# Supplementary material for: The Impact of Recombination on Nucleotide Substitutions in the Human Genome
Source: PLoS Genet. 2008 May 9;4(5):e1000071. doi: 10.1371/journal.pgen.1000071 (PMC2346554; doi:10.1371/journal.pgen.1000071)
Supplement: Text S1 — Correlations between the stationary GC-content and the current GC-content, the crossover rate and distance to telomeres. (0.05 MB PDF) [file pgen.1000071.s005.pdf]

Correlations between the stationary GC-content and the current GC-content, the crossover rate and distance to telomeres.

Data set: 1 Mb genome tiling

GC*: stationary GC-content

GC: current GC-content

CO: crossover rate (HapMap data)

LDT: Log distance to telomere in bp

GC* *vs* (GC) :	$R^2 = 0.25$
GC* *vs* (LDT) :	$R^2 = 0.35$
GC* *vs* (CO) :	$R^2 = 0.36$
GC* *vs* (GC, LDT) :	$R^2 = 0.42$
GC* *vs* (GC, CO) :	$R^2 = 0.44$
GC* *vs* (CO, LDT) :	$R^2 = 0.47$
GC* *vs* (GC, CO, LDT) :	$R^2 = 0.51$
