# Supplementary material for: The Impact of Recombination on Nucleotide Substitutions in the Human Genome
Source: PLoS Genet. 2008 May 9;4(5):e1000071. doi: 10.1371/journal.pgen.1000071 (PMC2346554; doi:10.1371/journal.pgen.1000071)
Supplement: Text S2 — Explicit definition of the matrix Q(3) defined in the Materials and Methods section. (0.18 MB PDF) [file pgen.1000071.s006.pdf]

Explicit definition of the matrix $Q^{(3)}$

The matrix $Q^{(3)}$ defined in Methods section takes the form

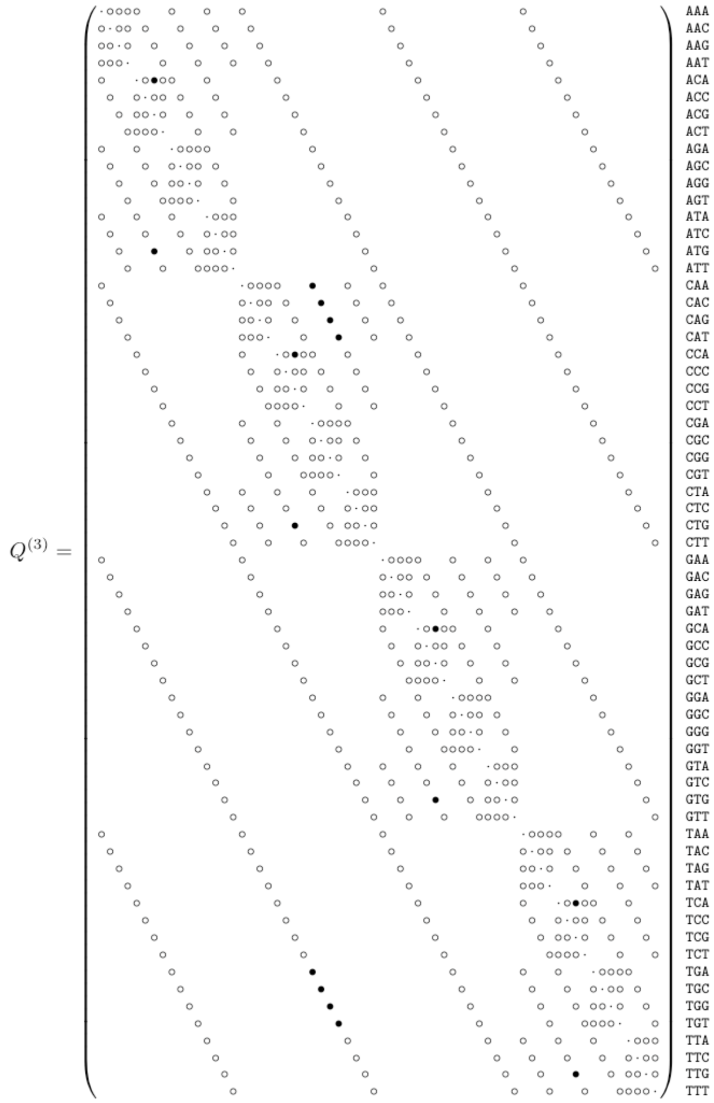

where an open circle '∘' denotes a non-vanishing matrix entry due to neighbor independent nucleotide substitution, e.g., $Q^{(3)}_{ACT,AGT} = q_{CG}$, and a filled circle '•' denotes an entry due to a neighbor dependent substitution process, e.g. $Q^{(3)}_{ACA,ACG} = q_{AG} + q_{CpG}$. The diagonal elements '·' have to be chosen such that all elements in one column add up to zero. All other elements are zero. Rows and columns are labeled by triplets of nucleotides from AAA to TTT as indicated.
