# Supplementary material for: The Impact of Recombination on Nucleotide Substitutions in the Human Genome
Source: PLoS Genet. 2008 May 9;4(5):e1000071. doi: 10.1371/journal.pgen.1000071 (PMC2346554; doi:10.1371/journal.pgen.1000071)
Supplement: Text S3 — Tests of the MCML algorithm using synthetic sequence data. (0.10 MB PDF) [file pgen.1000071.s007.pdf]

Supplementary Material

The Impact of Recombination on Nucleotide Substitutions in the Human Genome

Laurent Duret, Peter F. Arndt

A new method to compute substitution rates: tests using Synthetic Sequence Data

To test and validate the method that we developed to compute substitution rates, we first applied it to synthetically generated sequence data. For this purpose we generated a 1 Mb-long ancestral sequence $\vec{\alpha}^0$ with uniform base composition and subsequently computed descendant daughter sequences using a stochastic algorithm to introduce nucleotide substitutions according to a predefined substitution model with known substitution frequencies. This has been done in accordance with a predefined phylogenies.

Here we present two test setups with phylogenetic trees as shown in Figures S1 and S2. In the first case we have 5 leaf nodes and different substitution frequencies along the branches: the four transversions processes occur with same frequencies in each branch, but vary from one branch to another (as indicated by the branch lengths in Figure S1), in addition the transition frequencies vary from one branch to another and introduce different GC biases (see the stationary GC content, GC*, in Table S1). All substitution models also included the neighbor dependent CpG methylation deamination process. The second test is closer to the actual situation mentioned in the main text, i.e. we used substitution frequencies comparable to those measured from human, chimp, and macaque alignments. The GC bias is the same in all branches and the outgroup is connected to the two sister species along long branches (see Figure S2). In addition we introduced gaps into the final sequences at the leaf nodes and subsequently removing the gapped sites lost 10% of the available sequence data, also disrupting CpG sites.

After generation of these sequences, we supplied only the sequences $\vec{\alpha}^i$ attached to the leaf nodes to the MCML algorithm and iteratively generated ancestral sequences and estimated

substitution frequencies. After recursively initializing the sequence at internal nodes with the consensus sequence of the leaf sequences, the algorithm converges after about 40 iterations corresponding to about 2h CPU time.

The described tests were repeated 500 times. In Tables S1 and S2 we summarize the results by providing the substitution frequencies, which have been used to generate sequences, along with the estimated frequencies (mean and standard deviations of the 500 tests are given). Apart for the two branches connected to the root node, i.e. branches labeled (0,x), all deviations of the estimated from the used frequencies are below 2%, standard deviations are generally of the order of 5% of the estimated values. On top of the substitution frequencies we are also able to reconstruct the ancestral GC content of sequences on internal nodes excluding the root node (last column in Tables S1 and S2).

We extensively performed further tests allowing for different nucleotide compositions and non-trivial di-nucleotide distributions in the ancestral sequence or taking a piece of human genomic sequence as an ancestral sequence. In addition we also generated synthetic sequences according to more complicated phylogenetic trees. All these tests clearly demonstrate the validity of our method and the results show basically the same behavior with respect to the deviations from the generating frequencies as presented in Tables S1 and S2.
